# DRA-Net: Medical image segmentation based on adaptive feature extraction and region-level information fusion

**DOI:** 10.1038/s41598-024-60475-y

**Published:** 2024-04-27

**Authors:** Zhongmiao Huang, Liejun Wang, Lianghui Xu

**Affiliations:** School of Computer Science and Technology, Xinjiang University, Urumqi, 830046 China

**Keywords:** Cancer, Medical research

## Abstract

Medical image segmentation is a key task in computer aided diagnosis. In recent years, convolutional neural network (CNN) has made some achievements in medical image segmentation. However, the convolution operation can only extract features in a fixed size region at a time, which leads to the loss of some key features. The recently popular Transformer has global modeling capabilities, but it does not pay enough attention to local information and cannot accurately segment the edge details of the target area. Given these issues, we proposed dynamic regional attention network (DRA-Net). Different from the above methods, it first measures the similarity of features and concentrates attention on different dynamic regions. In this way, the network can adaptively select different modeling scopes for feature extraction, reducing information loss. Then, regional feature interaction is carried out to better learn local edge details. At the same time, we also design ordered shift multilayer perceptron (MLP) blocks to enhance communication within different regions, further enhancing the network’s ability to learn local edge details. After several experiments, the results indicate that our network produces more accurate segmentation performance compared to other CNN and Transformer based networks.

## Introduction

With the rapid development and progress of medical imaging technology, computer-aided diagnosis (CAD) is expected to help doctors reduce workload and improve work efficiency^1–5^. And medical image segmentation is a key step in CAD. Its task is to accurately identify the target organ, tissue, or lesion area from a given medical image. So it is of great significance in evaluating diseases, planning treatment strategies, and monitoring disease progression. But as a result of imaging equipment quality and technology limitations, medical images^6,7^ compared with the other images often have lower contrast and more noise. For example, colon tissue images can be diagnosed by observing and judging stained histopathological samples through a microscope. The tissue images recorded by microscopes are not only noisy but also have the characteristics of complex tissue texture and fuzzy boundaries that are not easy to distinguish. Therefore, it is essential to find a method that can accurately segment medical images.

In recent years, deep learning methods have achieved great success in different tasks^8^. In medical image segmentation tasks, compared to traditional algorithms, deep learning based algorithms^9–12^ can automatically extract features, which effectively overcomes the shortcomings of traditional medical image segmentation algorithms that need to design manual features and rely too much on the prior cognition of medical experts. Moreover, deep learning algorithms are highly transferable and can be rapidly extended to different tasks with the help of transfer learning. Among the deep learning algorithm model based on convolutional neural network (CNN), U-Net^13^ is a representative work. And many subsequent medical image segmentation works are based on the idea of this model. Such as U-Net++^14^, U-Net3+^15^, V-Net^16^, Res-UNet^17^, Y-Net^18^, ARU-Net^19^. Although CNN has become the mainstream method for medical image segmentation, it also has weak points because of ignoring the relationship between long-distance context information.

With the continuous development of deep learning algorithms, some researchers have introduced well-performed methods from natural language processing (NLP)^20,21^ to vision field, such as self-attention mechanism^21^. For its powerful global modeling ability, Transformer achieve good results in machine translation tasks. Later, the outcomes of ViT^22^ in image classification also confirmed that the self-attention mechanism can be used in the field of visual processing. Although ViT and other works^23–25^ have proved the effectiveness of the self-attention mechanism, it needs the support of a large amount of data due to the lack of inductive biases. Applying it to small amounts of medical images cannot fully utilize its advantages, and the calculation cost is relatively large. Lately, new progress has been made in the research of multilayer perceptron (MLP) model^26–28^. The results of MLP-Mixer^29^, AS-MLP^30^ and other works can also be comparable to the models based on CNN or self-attention mechanism. MLP, like self-attention mechanism, has global modeling ability and has strong performance under small model size. Still when the model size is enlarged, it may influence the model effect due to overfitting^31^.

Based on the above issues, we propose a medical image segmentation model based on adaptive feature extraction and regional level information fusion in this paper. This model can aggregate attention into different dynamic regions based on similarity measurement. Because we note that the size of the target area in medical images is variable, and using only local fixed structures to extract features may lead to information loss. So we propose a dynamic regional attention module, which can measure the similarity of various features and divide similar features into one region to achieve the goal of automatically selecting different modeling ranges based on features. It is equivalent to delineating different regions on the feature map and fusing information within different regions, enabling the network to extract more surrounding feature information. Finally, we also use the ordered shift MLP module to rearrange features, moving the channels of feature maps from different spatial directions to obtain information flow, thereby enhancing communication between feature groups that have learned different features. By combining these parts, we can achieve multi-range feature interactions, improve the network’s ability to learn local details and reduce information loss. In summary, the contributions of this paper include the following three points:

1. We design a dynamic regional attention module that can measure the similarity between features and divide similar features together to form explainable local dynamic regions. Through this approach, similar regions complement each other and dissimilar regions are excluded. At the same time, attention within the dynamic region is utilized to facilitate interactive fusion of features and reduce information loss.
2. We also design a ordered shift MLP module. This module rearranges features through feature selection, divides different features into different feature groups, and promotes local communication between different features through spatial displacement, thereby improving the network’s ability to extract local details.
3. Different from CNN or Transformer which adopts local fixed or global feature extraction, this paper designs two different efficient feature extraction strategies and combines them to propose a medical image segmentation network based on hybrid encoding and decoding, achieving advanced segmentation results on different datasets.

## Related work

### Model based on CNN

In the past few years, much of the work has been based on improvements to CNN models. U-Net is a representative work. It realizes the feature fusion of different levels through long skip connections and improves the segmentation accuracy. The subsequent U-Net++ further improved the multi-layer feature fusion mode to enhance the feature fusion effect. Milletari et al, extended the U-Net model to three dimensions and preserved more details by adding short skip connections in ResNet^32^ at each stage of the down-sampling. These models can prove that the local modeling function of CNN is crucial. However, increasing the receptive field during feature extraction may provide assistance in model segmentation performance.

### Model based on transformer

With the success of Transformer in the visual field, Some scholars have attempted to apply it to medical image segmentation tasks^33,34^. Swin-Unet^35^, a medical image segmentation model based on pure Transformer appears. It uses hierarchical Swin-Transformer^36^ with shifted Windows as an encoder to extract contextual features, which allows it to learn the interaction of global and remote semantic information. But medical images have a strong local structure, completely ignoring this locality is not advisable. Then, TransUNet^37^ integrates CNN and Transformer to design a better method, so that the global context encoded by Transformers can be combined with detailed high-resolution spatial information from CNN features to achieve accurate positioning. TransFuse^38^ runs CNN and Transformer in parallel. As a result, global information can be captured without building deep networks, while maintaining sensitivity to low-level context. Zhu et al.^39^ propose a brain tumor segmentation method based on the fusion of deep semantics and edge information in multimodal MRI. Its designs semantic segmentation module that uses Swin-Transformer as the backbone, which can reduce computational complexity and achieve efficient dense prediction. X-Net^40^ combines CNN and Transformer to interactively fuse local and global information during encoding, achieving better segmentation results. Similarly, Liu et al.^41^ also demonstrated the importance of properly integrating CNN and Transfomer for extracting global and local information in retinal segmentation tasks. Although using Transformer is a new idea to solve the problem of medical image segmentation, the model based on Transformer will not perform well when training data is insufficient.

### Model based on MLP

While MLP is not a new concept, a lot of new work based on MLP is appearing. For example, MLP-Mixer does not use CNN and Transformer, and uses two different MLP layers repeatedly to realize the interaction of spatial position and channel information, for obtaining spatial position and channel characteristics. In addition, AS-MLP puts forward a new way of thinking based on MLP. It captures local dependencies by acquiring information flows from different axial directions through moving the channel of feature maps. It is able to implement the same local receptive field as the CNN class architecture when using pure MLP architecture.

In summary, CNN is still the best choice for segmenting small data volume medical datasets. However, using it alone for feature extraction of medical images has certain limitations. So after analyzing existing methods, this paper proposes a new feature extraction and information fusion method. On the one hand, it fully utilizes local correlations in medical images and enhances communication between similar feature regions. On the other hand, it combines dynamic regional attention module (DRA) and ordered shift MLP in a parallel manner for multi-feature fusion, achieving higher segmentation performance in medical images.

## Method

As mentioned above in the introduction, the organization and texture of medical images are complex and the boundary is blurred, which makes the identification of medical images a very complicated and time-consuming work. Therefore, the data volume of medical image dataset is smaller than other datasets. Moreover, medical images have strong local structure. Compared with CNN, Transformer structure treats all tokens equally and ignores locality. In view of this situation, we used a small model with CNN as the backbone and integrating ordered shift MLP module and dynamic regional attention module.

**Figure 1:**
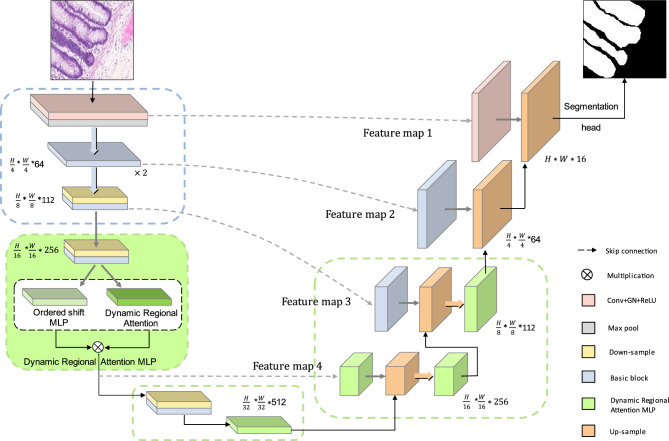
Overall network architecture.

The overall architecture of the network is shown in Fig. 1, which consists of three parts. The first is the encoder, considering that the target size in medical images is usually dynamic and variable, we not only use convolution operation to extract feature information in the encoder, but also design the ordered shift MLP module to promote local information exchange and the dynamic regional attention module to increase the receptive field during feature extraction. The second part is the decoder, which restores the feature resolution of the encoded feature through bilinear interpolation algorithm to predict the target region. The third part is the skip connection. When the decoder is working, the same stage features of the encoder and decoder end are fused by the skip connection to prevent the information loss that will occur in the target area from being predicted directly using the coding features.

### Encoder

#### CNN part

**Figure 2:**
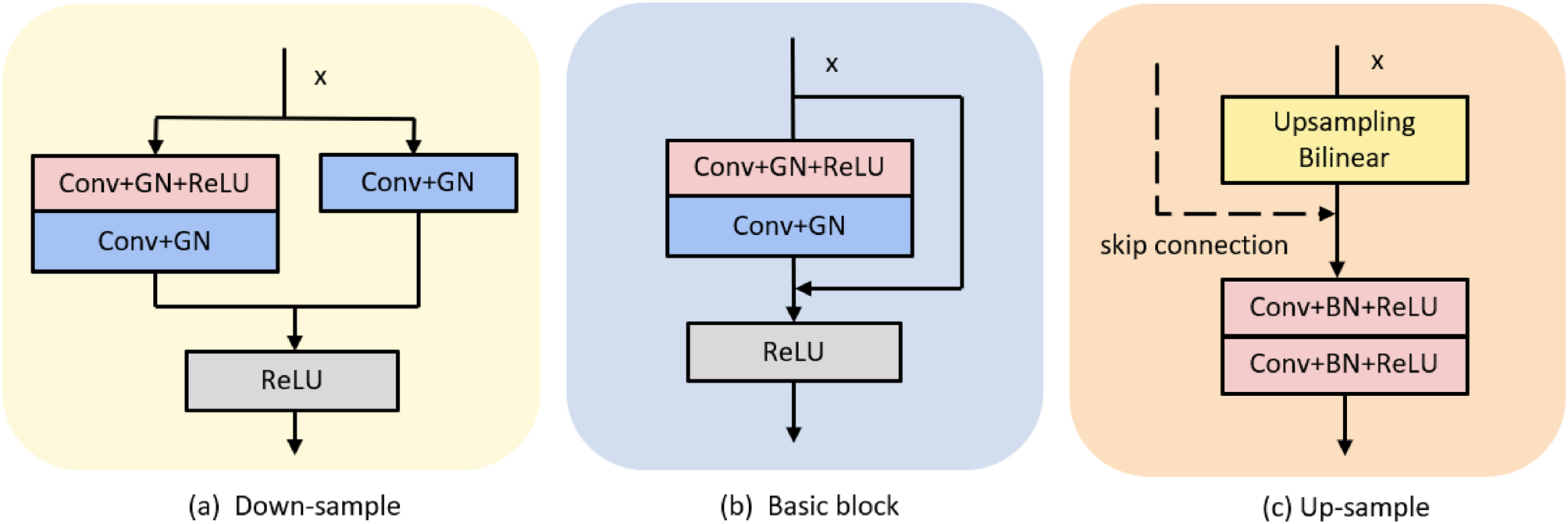
Specific illustration of network architecture. (**a**) Structure of Down-sample. (**b**) Structure of Basic block. (**c**) Structure of Up-sample.

We set the input image size to $224\times 224$. The specific details of the network architecture are shown in Fig. 2a,b, shallow texture features and deep abstract features can be available by the encoder. The five stages in the down-sampling imitate the method of extracting features from ResNet34^32^. Considering the amount of data, we set the number of layers in the second to fifth stages to 2. By this means, we can avoid overfitting and waste of computing resources. In the down-sampling process, we adopt group normalization^42^. In contrast to batch normalization, group normalization groups channels and normalizes them within each channel group so that batch size does not affect the model.

#### Ordered shift MLP module

**Figure 3:**
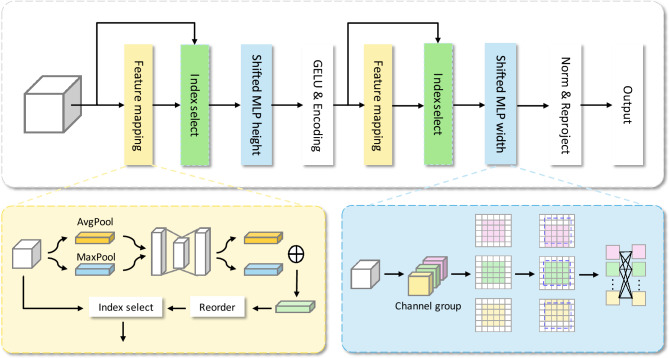
Overview of the proposed ordered shift MLP module.

In this section, we will introduce the ordered shift MLP module. The specific details of the module are shown in Fig. 3. Assume that the input feature dimension is $X \in {\mathscr {R}}^{H \times W \times C}$. First, we will extract the spatial feature mapping information by performing average pooling and max pooling on the input features. The feature mapping information reflects the ordering relationship between the input feature channels. And use the reordered feature mapping information $S_1 \in {\mathscr {R}}^{c \times 1 \times 1}$ to order the input features to get the sequential feature $X_S \in {\mathscr {R}}^{H \times W \times C}$.

1 $$\begin{aligned} {S_1}= & {} S\mathrm{{ort}}(MLP(AvgPool(X)) + MLP(MaxPool(X))). \end{aligned}$$

2 $$\begin{aligned} {X_S}= & {} Index\_selected(X,{S_1}). \end{aligned}$$

Next, we’re going to padding $$X_S$$ to obtain $$X_{Spad}$$, then group the filled features in the channel dimension. Let $${X_S} = {[{X_S^ 1}, \ldots , {X_S^g}]}$$, where g is the number of groups. Let i represent any set of feature maps, then $${X_S^i} \in {R^{H*W*\frac{C}{g}}}$$. The communication between local information is obtained by shifting channel blocks belonging to different groups in the height direction. The local information flow obtained after moving the grouping is represented by $${T_H} \in {\mathscr {R}}^{H \times W \times C}$$.

3 $$\begin{aligned} {T_H} = ShiftH({X_{Spad}}). \end{aligned}$$

At the same time, we use a depthwise separable convolution operation^43^ to encode the position information in the MLP layer. As shown in formula (4).

4 $$\begin{aligned} {\text {T}}=GELU{\left( {DWConv{\left( {MLP{\left( {T_H}\right) }}\right) }}\right) .} \end{aligned}$$

Similarly, different local information flows can be obtained in the width direction to obtain receptive field that are different from convolution operations. For feature T, we use the rearranged feature mapping information $S_2 \in {\mathscr {R}}^{c \times 1 \times 1}$ to obtain the ordered feature $T_S \in {\mathscr {R}}^{H \times W \times C}$.

5 $$\begin{aligned} {S_2}= & {} S\mathrm{{ort}}(MLP(AvgPool(X)) + MLP(MaxPool(X))). \end{aligned}$$

6 $$\begin{aligned} {T_S}= & {} Index\_selected(T,{S_2}). \end{aligned}$$

We also consider the sequential feature $T_S$ performs a series of operations such as filling, channel grouping, etc. The final output $X_Output \in {\mathscr {R}}^{H \times W \times C}$ is obtained.

7 $$\begin{aligned} {T_W}= & {} ShiftW({T_{Spad}}). \end{aligned}$$

8 $$\begin{aligned} {X_{\mathrm{{output}}}}= & {} MLP({T_w}). \end{aligned}$$

Our proposed ordered shift MLP module obtains surrounding feature information through channel grouping shift, enhancing local information exchange. Before grouping, we sort the features, so that more local communication can be obtained between the feature groups that have learned different features in the subsequent process.

#### Dynamic regional attention module

**Figure 4:**
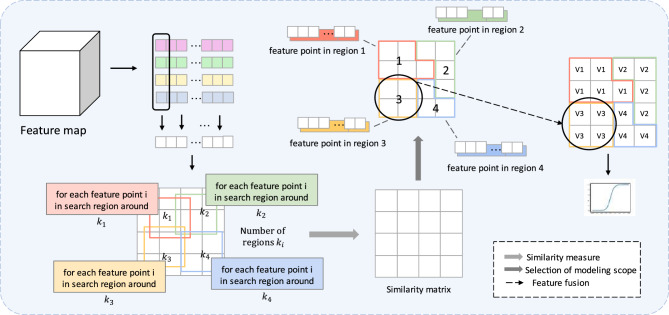
Working Principle of dynamic regional attention MLP.

This section will introduce our proposed dynamic regional attention module. This module is used to measure feature similarity and select different modeling ranges for feature fusion. Figure 4 shows the changing process of the input feature map. Assume that the input feature dimension is $X \in {\mathscr {R}}^{H \times W \times C}$, where H represents the height of feature map, W represents the width of feature map, and C represents the number of feature map channels. During information fusion, this method fuses each spatial information of the input feature map into a channel through a fully connected layer to obtain one-channel feature map. Through this operation, we hope to synthesize the previously extracted features and get all the possibilities of each spatial feature. After passing through the fully connected layer, we perform dimension transformation on the features to generate feature map $X_{fc} \in {\mathscr {R}}^{H \times W \times 1}$.

9 $$\begin{aligned} {\text {X}}_{{\text {fc}}}=Reshape{\left( {FC{\left( {Flatten{\left( {X}\right) }},1\right) }},h,w\right) }. \end{aligned}$$

##### Similarity measure

Next, we calculate the similarity between each feature point and classify the feature points on the one-channel feature map. In this way, we can divide similar features in the feature map together, which is equivalent to defining a boundary for similar features. In the module, we set the number of different regions to k. Let $X_{fc}^i$ represents the feature value corresponding to the i-th feature point on the feature map. $h \in {\mathscr {R}}^{\left[ 0,H-1 \right] }$ represents the coordinate of the feature value in the height direction, while $w \in {\mathscr {R}}^{\left[ 0,W-1 \right] }$ represents the coordinate of the feature value in the width direction. In the process of division, the module will record the corresponding spatial coordinates and feature values of each feature point. Because the category of feature points is judged in the search area according to these two points. The calculation formula is shown in the following formulas (7–9).

The similarity measure S is determined by feature proximity $d_f$ and spatial proximity $d_s$. Feature proximity is calculated by the Euclidean distance between the value of feature point in the region and the value of central point, as shown in formula (7).

10 $$\begin{aligned} {\text {d}}_{{\text {f}}}={\sqrt{\left( {X_{fc}^i-X_{fc}^j}\right) ^2}}. \end{aligned}$$

Similarly, the spatial proximity is calculated by the Euclidean distance between the spatial coordinate values of feature points and the spatial coordinate values of central points in the region, as shown in formula (11).

11 $$\begin{aligned} {\text {d}}_{{\text {s}}}={\sqrt{\left( {h_i-h_j}\right) ^2+\left( {w_i-w_j}\right) ^2}}. \end{aligned}$$

In order to combine the two approaches into a similarity measure S, it is necessary to normalize the feature proximity and spatial proximity through m and s. Where m is a constant to determine the spatial distance weight ratio, and s is determined according to the specific number k of dividing feature map.

12 $$\begin{aligned} {\text {S}}={\sqrt{\left( \frac{d_f}{m}\right) ^2+\left( \frac{d_s}{s}\right) ^2}}. \end{aligned}$$

##### Modeling scope selection

**Figure 5:**
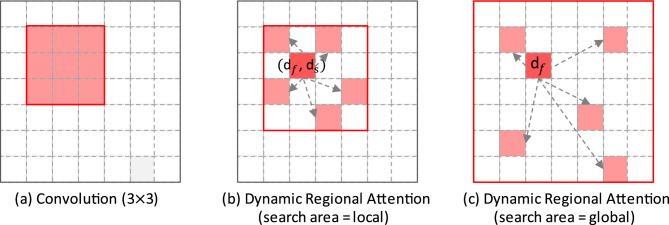
Comparison of receptive fields between convolution and DRA module. (**a**) Convolutional Kernel (3*3), (**b**) The first methods, similarity measure is based on the feature proximity $d_f$ and spatial proximity $d_s$, and search area is not global. (**c**) The second methods,similarity measure is only based on the feature proximity $d_f$, and search area is global.

We designed two methods based on similarity measurement and search area. The first methods, similarity measure is based on the feature proximity $d_f$ and spatial proximity $d_s$, and search area is not global; The second methods, similarity measure is only based on the feature proximity $d_f$, and search area is global. After the search is completed, the feature map will be divided into k non overlapping regions. The receptive fields comparison of two different search methods and convolution is shown in Fig. 5. The red box represents the receptive field.

##### Feature fusion

After selecting the appropriate modeling range, we will perform feature fusion on features in different regions. Assuming $k_{i}$ regions are set, it means that the feature map $X_{fc} \in {\mathscr {R}}^{H \times W \times 1}$ will be divided into $k_{i}$ blocks. Let $x_{i}$ represent the feature area corresponding to center $k_{i}$, and $x_{i}^j$ represent the feature value corresponding to the j-th feature point in the i-th region. Then there is $[x_{1},x_{2},...,x_{i}] \in X_{fc}$, $[x_{1}^0,x_{1}^1,...,x_{1}^{n-1}] \in x_{1}$. So it can be represented by formula 13.

13 $$\begin{aligned} {\text {x}}_{{\text {i}}}= \frac{1}{n} {\left( {\sum _{j=0}^{n-1}x_{i}^j}\right) }. \end{aligned}$$

Output of dynamic regional attention module $M_{DRA}\left( X\right) \in {\mathscr {R}}^{H \times W}$ can be represented by the following formula (14). Where SM represents similarity measure, MSS represents Modeling scope selection, and FF represents feature fusion. Finally, as shown in formula (15), we combine it with the output of the ordered shift MLP block to achieve the goal of increasing feature interaction within the region.

14 $$\begin{aligned} {\text {M}}_{{\text {DRA}}}\left( {\text {X}}\right)= & {} {sigmoid(FF(MSS\left( SM\left( X_{fc}\right) \right) ))}. \end{aligned}$$

15 $$\begin{aligned} {\text {X}}^{'}= & {} {{M_{DRA}\left( X\right) } \otimes MLP\left( X\right) }. \end{aligned}$$

The local correlation of medical images is relatively strong, and we need to use this prior knowledge to help the network segment medical images better. However, only using convolution operations to extract features can result in the loss of some key information, so this paper proposes two different methods for feature extraction. Both of these methods can increase the network’s ability to extract similar feature information. The difference is that the first method can extract feature information far away from the feature point, while the second method can extract feature information within a certain range around the feature point. Different from other methods^44–47^, The perception area of this module during the selection of regions and feature fusion process is adaptive and determined by similar features. In this way, the module can fully utilize all feature information to improve the learning ability of the network.

### Decoder

In the decoder, we will use the deep feature map through multiple up-sampling stages to predict the segmentation map. The up-sampling part utilizes bilinear interpolation algorithm to obtain feature maps of different layers, as shown in Fig. 2c. The same level feature maps of up-sampling and down-sampling are concatenated together through long-distance skip connections to effectively preserve some feature details.

### Loss function

In this paper, the loss combination of binary cross entropy (BCE) and Dice are used in the loss section. The specific formula is as follows:

16 $$\begin{aligned} {\text {Loss}}_{{\text {BCE}}}= & {} {-w_n\left[ y_n \cdot log_{}{x_n} + (1-y_n) \cdot log{}{(1-x_n)}\right] }. \end{aligned}$$

17 $$\begin{aligned} {\text {Loss}}_{{\text {Dice}}}= & {} {1- \frac{2\sum _{i}x_iy_i}{\sum _{i}x_i + \sum _{i}y_i}}. \end{aligned}$$

18 $$\begin{aligned} {\text {Loss}}_{{\text {total}}}= & {} {0.5*Loss_{BCE} + Loss_{Dice}}. \end{aligned}$$

The loss function shown in formula (18) combines BCE loss and Dice loss. In the BCE loss formula, $x_n$ represents the element value in the prediction diagram, and $y_n$ represents the corresponding element value in the label diagram. Parameter $w_n$ means that we can manually rescale the loss weight for each element, and other parameters such as reduction were not specified in the training. In the Dice loss formula, $x_i$ is the probability value that the i-th element in the prediction diagram belongs to a prospect of a certain category, and $y_i$ is the true value of the i-th element in the label diagram. Dice loss, unlike BCE loss, is not affected by foreground size, and BCE loss can play a guiding role in Dice loss during network learning. Therefore, it is more reasonable to combine the two losses for network learning.

## Experiments and results

### Datasets

**Figure 6:**
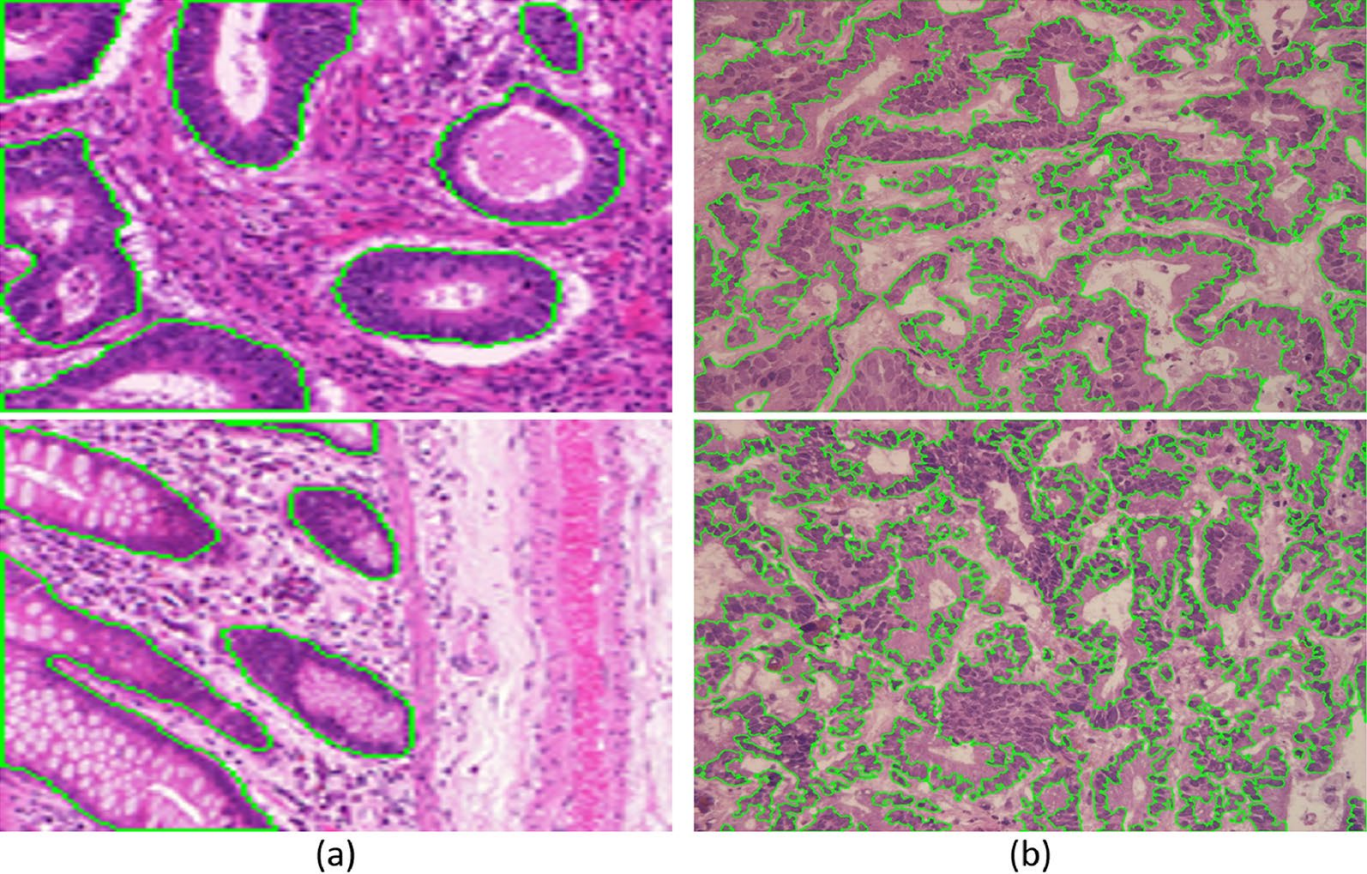
The difference between GlaS and CoCaHis. (**a**) Two images of GlaS, (**b**) Two images of CoCaHis.

We used two medical datasets GlaS^48^ and CoCaHis^49^ to validate the method. The specific information of the dataset is as follows: The GlaS dataset consists of 165 images from 16 H &E staining histological sections of colorectal adenocarcinoma in T3 or ${T4}^2$ stages, and each section belongs to different patients. The CoCaHis dataset contains the microscopic images of 82 H &E stained sections, which are frozen samples of liver metastatic colon cancer collected from 19 patients during surgery. In colorectal cancer, the key criteria for cancer grading are the morphology of the intestinal glands including architectural appearance and gland formation. So the foreground information in GlaS is fragmented but not complex. However, CoCaHis contains images of colon cancer metastasis, in which the cancer cells are irregularly arranged. The foreground information of the dataset is not only scattered but also complicated. Figure 6 shows the difference between the two datasets. In GlaS, we used 85 images for training and 80 images for test. And in CoCaHis, we use 65 images for training and 17 images for test.

### Implementation details

We conduct our experiment on Python 3.6 and torch 1.8.1. NVIDIA Tesla V100 GPUs are used in training and testing models. The initial learning rate of the model is set to 0.001. During the training process, the cosine annealing method is used to attenuate the learning rate to the minimum value of 0.00001. The default optimizer is Adam. We set the weight decay to 0.0001. In the experiment, the batch size is set to 4, the number of training epochs is 400, and the input image size is 224 * 224. At the same time, the image will be rotated, flipped, contrast enhanced, and so on.

### Evaluation metrics

In this paper, we adopt two evaluation indicators, IoU (intersection over union) and Dice (dice similarity coefficient), to measure the similarity between the prediction diagram and the label diagram. The formula is as follows.

19 $$\begin{aligned} {\text {IoU}}= & {} {\frac{TP}{FP + TP + FN}}, \end{aligned}$$

20 $$\begin{aligned} {\text {Dice}}= & {} {\frac{2TP}{FP + 2TP +FN}}, \end{aligned}$$

21 $$\begin{aligned} {\text {Recall}}= & {} {\frac{TP}{TP + FN}}, \end{aligned}$$

22 $$\begin{aligned} {\text {Specificity}}= & {} {\frac{TN}{TN + FP}}, \end{aligned}$$

23 $$\begin{aligned} {\text {Precision}}= & {} {\frac{TP}{FP + TP }}. \end{aligned}$$

where TP is True Positive, that is, the correct foreground area in the prediction diagram. TN is True Negative, which is the correct background area in the prediction diagram. FP is False Positive and is the part judged as foreground area in the prediction diagram but as background area in the label diagram. FN is False Negative and is the portion of the prediction diagram that is judged to be a background region and the label diagram that is judged to be a foreground region.

### Comparative experiment

To prove the segmentation performance of the proposed model on these two datasets, We compared the final results with some advanced models, including CNN-based models U-Net, U-Net++, U-Net3+,Transformer based models MedT^50^, UCTransNet^51^, DAEFormer^52^, BiFormer^53^, UDTransNet^54^, ConvFormer^55^, and other medical segmentation models such as Attention U−Net^56^, UNeXt^57^. The results of comparative experiment are shown in Tables 1 and 2. It can be seen from the Tables that the method has certain effect improvement on these two datasets, and has certain advantages over the existing methods.

**Table 1:** Results compared with the most advanced models on GlaS. Bold represents the highest value and italic represents the second highest value bold.

Network	GlaS				
	IoU (%)	Dice (%)	Recall (%)	Specificity (%)	Precision (%)
U−Net^13^	76.84	86.30	86.19	87.21	87.91
U−Net++^14^	78.10	87.07	86.62	*88.82*	*89.93*
U−Net3+^15^	78.09	86.81	88.57	83.39	87.61
Attention U−Net^56^	77.53	86.98	89.78	83.39	85.20
UTNet^24^	80.30	88.16	**92.12**	82.98	86.55
MedT^50^	73.32	83.72	85.89	80.94	84.56
Swin−Unet^35^	78.83	65.93	81.01	75.40	79.00
UCTransNet^51^	*82.21*	*89.62*	90.10	88.59	89.73
TransUNet^37^	79.10	87.63	86.59	88.77	88.12
DAEFormer^52^	76.28	85.71	88.50	81.62	84.83
BiFormer^53^	85.52	75.67	87.25	81.94	86.07
UDTransNet^54^	89.45	81.73	90.47	87.52	89.77
ConvFormer^55^	86.42	77.00	*90.83*	77.40	83.81
UNeXt^57^	76.85	86.79	87.31	85.73	86.64
Ours	**82.45**	**90.23**	90.69	**89.42**	**90.11**

**Table 2:** Results compared with the most advanced models on CoCaHis. Bold represents the highest value and italic represents the second highest value bold.

Network	CoCaHis				
	IoU (%)	Dice (%)	Recall (%)	Specificity (%)	Precision (%)
U−Net^13^	63.24	75.20	75.90	87.53	*83.89*
U−Net++^14^	65.90	78.28	78.29	87.81	83.79
U−Net3+^15^	65.16	77.06	77.15	88.89	83.80
Attention U−Net^56^	60.49	71.91	69.65	**90.92**	**86.90**
UTNet^24^	61.67	74.88	83.59	84.19	71.90
MedT^50^	64.38	77.41	81.27	86.66	78.14
Swin−Unet^35^	51.77	65.20	66.95	84.49	78.60
UCTransNet^51^	63.43	76.67	84.74	80.92	73.82
TransUNet^37^	*67.48*	*79.84*	83.96	87.01	78.21
DAEFormer^52^	64.80	77.70	83.60	86.61	75.74
BiFormer^53^	74.59	61.41	81.53	82.96	74.80
UDTransNet^54^	77.32	64.22	82.05	84.17	76.19
ConvFormer^55^	76.09	63.08	80.61	83.36	75.52
UNeXt^57^	63.22	77.24	*84.45*	85.10	71.40
Ours	**70.24**	**82.45**	**86.95**	*88.90*	78.60

**Figure 7:**
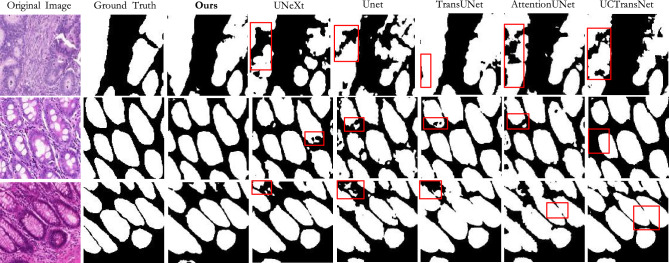
The qualitative comparison of different models on GlaS.

Results on GlaS: According to the results in Table 1, our method outperforms other methods in most indicators. It achieved an accuracy of 82.45$\%$ on the IoU and 90.23$\%$ on the Dice. Based on Table 1 and Fig. 7, it can be seen that the foreground information of this dataset is slightly scattered and the cell staining is different in depth. The U-Net has fewer layers and cannot fully learn these complex information, so the effect is poor. Compared to U-Net, U-Net++ and U-Net3+ increase communication at different levels, which helps to segment more accurate foreground regions. At the same time, most of the cancer cells have regular boundaries, so the existing medical image segmentation models have good segmentation results. And we use the ordered shift MLP module, which can enable more local communication between feature groups that have learned different features in feature extraction, reducing information loss. Therefore, our method produces fewer under segmentation cases and achieves higher results.

**Figure 8:**
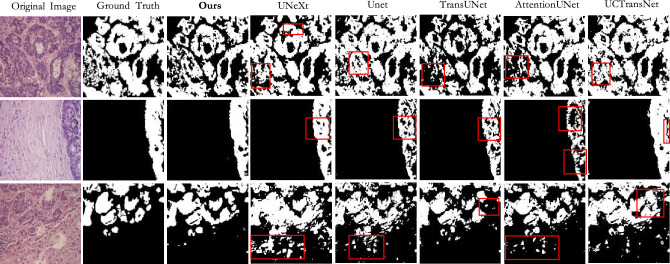
The qualitative comparison of different models on CoCaHis.

**Figure 9:**
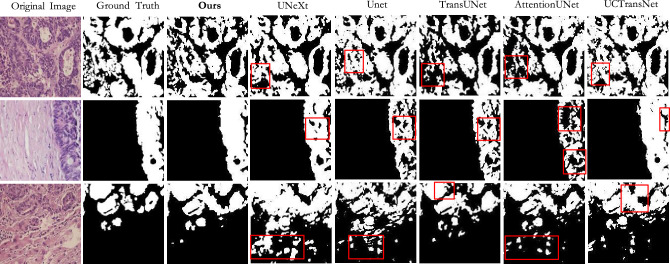
The qualitative comparison of differences in prediction diagram details between different models on CoCaHis.

Result on CoCaHis: According to the results in Table 2, our method outperforms other models in almost all indicators. It achieved an accuracy of 70.24$\%$ on the IoU metric and 82.45$\%$ on the Dice metric. Due to the complexity of the foreground information of this dataset, most of the existing methods have poor segmentation effect on it. Combined with Table 2 and Figs. 8 and 9, it can be seen that some Transformer-based models have lower effects than CNN-based models. This may be due to the small size of the dataset, so Transformer-based models cannot learn features from the limited data. Our method utilizes the dynamic regional attention module to cluster locally similar pixels, forming interpretable local regions. And using the attention in the super pixel to make the local information interaction, then reduce the information loss, so the effect is better. Most of the textures in this dataset are complex, and the corresponding boundary information is also abundant and complex. We need to reduce information loss to ensure the accuracy of model segmentation.

Figures 7, 8 and 9 shows the prediction diagrams generated by different models. From the figure, we can see that the proposed method produces better segmentation results than other models. Compared with the results of other models, it improves the edge information of some segmentation areas with incorrect prediction, especially some foreground parts, and makes the results closer to the label diagram. The red box in other prediction figures shows the poor accuracy of the model compared with the method in this paper. From the figure, we find that CNN-based models show more under-segmentation, because these models mainly use convolution operations in which the convolution kernel is locally fixed. It can not extract all the relevant feature information at once, which may lead to the loss of some key information. Transformer-based models have the ability to model globally, so it has fewer under-segmented parts compared to CNN-based models. However, they may blur some local edge details and cause over-segmentation. Unlike the above methods, our method adaptively selects different modeling ranges based on different features, and can extract similar feature information around it. And it can be seen that our method can perform more accurate segmentation while retaining detailed shape information in these prediction results.

To sum up, CNN-based models have more obvious advantages in segmentation compared to some Transformer-based models when dealing with small amounts of data. Due to the strong local structure of medical images, some features are often more closely related to surrounding features, so the ability of convolution operations to extract local information makes CNN-based models perform well in segmentation. Compared with CNN, Transformer pays more attention to global information, ignoring the importance of local information. Moreover, Transformer based models require the support of large amounts of data, so the segmentation effect is not good. Meanwhile, from Table 3, we can see that most Transformer-based models perform lower than CNN-based models in terms of model parameters and inference speed. However, our method adaptively selects the extraction range of local information, making up for the shortcomings of convolution operation in extracting feature information. It not only has better effect than the CNN-based model and the Transformer-based model, but achieves good levels in model parameters, inference speed, and other aspects.

**Table 3:** Comparison of performance with models based on different methods.

Network		Pramas (M)	GFLOPs	Inf. Time (ms)
Based on CNN	U−Net^13^	7.77	10.52	9.68
	U−Net++^14^	9.16	26.72	21.37
	U−Net3+^15^	26.97	152.87	68.63
	Attention U−Net^56^	34.88	51.02	12.65
Based on Transformer	UTNet^24^	10.01	13.15	92.43
	MedT^50^	1.60	21.24	1861
	Swin−Unet^35^	41.30	8.63	58.63
	UCTransNet^51^	66.22	32.87	1328
	TransUNet^37^	93.19	24.63	52.63
	DAEFormer^52^	29.61	25.95	92.33
	BiFormer^53^	87.46	49.63	262.58
	UDTransNet^54^	33.80	26.51	107.99
	ConvFormer^55^	115.61	92.74	64.31
Based on MLP	UNeXt^57^	0.25	0.08	10.93
	Ours	15.28	4.15	69.88

### Ablation studies

In this section, we conducted multiple sets of experiments to verify the specific role of the proposed module, and conducted ablation analysis on the GlaS and CoCaHis datasets. Firstly, We are based on U-Net. Then, we add ordered shift MLP module and dynamic regional attention module respectively to U-Net. Meanwhile, we also validated the parameters and inference speed, as shown in Table 6.

From Tables 4 and 5, it can be seen that adding ordered shift MLP and dynamic regional attention has an improving effect on segmentation accuracy. Proving that increasing the receptive field during feature extraction is crucial for reducing information loss. In addition, we also provided a Visualization of the ablation studies (Table 6).

**Table 4:** Results of ablation analysis on the GlaS dataset. Bold represents the highest value and italic represents the second highest value bold.

Method	GlaS				
	IoU (%)	Dice (%)	Recall (%)	Specificity (%)	Precision (%)
U−Net	76.84	86.30	86.19	87.21	87.91
Conv stage	79.83	88.64	88.31	89.23	89.45
Conv stage+MLP	*81.67*	*89.80*	89.93	*89.35*	*89.99*
Conv stage+DRA	81.59	89.75	**91.23**	87.79	88.63
Conv stage+DRA+MLP	**82.45**	**90.23**	*90.69*	**89.42**	**90.11**

**Table 5:** Results of ablation analysis on the CoCaHis dataset. Bold represents the highest value and italic represents the second highest value bold.

Method	CoCaHis				
	IoU (%)	Dice (%)	Recall (%)	Specificity (%)	Precision (%)
U−Net	58.71	71.10	67.13	**90.87**	**87.72**
Conv stage	67.50	80.35	84.90	87.50	76.78
Conv stage+MLP	67.71	80.61	85.28	88.07	76.72
Conv stage+DRA	*68.36*	*81.06*	**87.02**	87.08	76.19
Conv stage+DRA+MLP	**70.24**	**82.45**	*86.95*	*88.90*	*78.60*

**Table 6:** Comparison of model performance by adding different modules.

Network	Pramas (M)	GFLOPs	Inf. Time (ms)
U−Net	7.77	10.52	9.68
Conv stage	14.32	2.36	18.28
Conv stage+MLP	15.27	2.46	31.18
Conv stage+DRA	14.33	4.14	60.78
Conv stage+DRA+MLP	15.28	4.15	69.88

**Figure 10:**
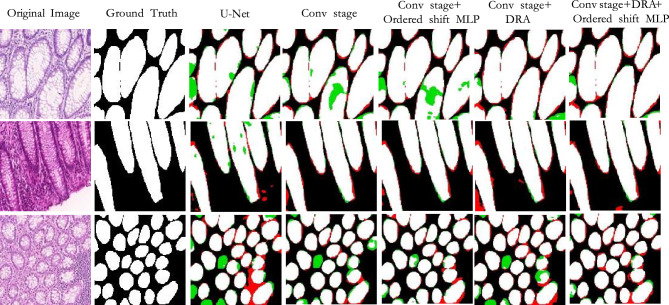
The ablation studies results of the GlaS, with red indicating the highest value and blue indicating the second highest value.

As can be seen from Fig. 10, for cell areas with lighter staining, the original U-Net is prone to under-segmentation, that is, it will mistakenly distinguish the light-colored areas in the foreground information as background information, resulting in the reduction of segmentation accuracy. However, the addition of DRA can effectively reduce under-segmentation, because DRA uses similarity measurement to form local interpretable regions, and increases local communication within the region, weakening the information loss caused by feature extraction only with convolution. With the DRA, this occurrence is reduced and the edge details of the foreground information are segmented more precisely. At the same time, for cells with blurred boundaries, U-Net cannot accurately judge the boundary details. After the application of the ordered shift MLP, the adhesion between the foreground area and the surrounding unrelated area can be effectively reduced, and the segmentation result can be significantly improved.

**Figure 11:**
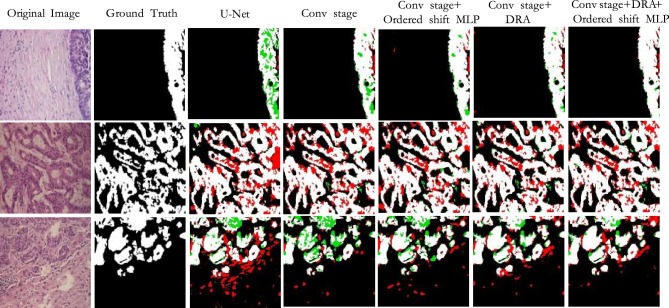
The ablation studies results of the CoCaHis, with red indicating the highest value and blue indicating the second highest value.

As can be seen from the experimental results in Fig. 11, U-Net is prone to over-segmentation for areas with blurred boundaries and normal cells, that is, it mistakenly identifies other tissue areas as cancer cells. Moreover, due to the complexity of cancer cell arrangement, the foreground region has rich edge information, and it will also lead to the expansion of the edge when the feature fusion. The convolutional layer setup in the baseline can effectively reduce this situation. At the same time, it is obvious from the fifth column that adding the ordered shift MLP can significantly reduce the phenomenon of under-segmentation. And through the subdivision of different feature areas through DRA, the boundary of the target area can be defined, and the finer details of the foreground area can be predicted. Therefore, the proposed module can be applied to the network at the same time to make the prediction graph closer to the real label.

#### Results of different search methods on DRA

Due to the similarity measure determining the degree of similarity between features, and the search range affecting the results of feature fusion, we chose two different methods for experiments to confirm the effectiveness of the method. The first, similarity measure is only determined by feature proximity, and the search area is global; The second, similarity measure is determined by both feature proximity and spatial proximity, and the search area is not global.

The comparison results are shown in Table 7. From the table, it can be seen that the second method yields better results. This further confirms that the local structure in medical images is strong, and adding this prior condition to distance measurement and search range can improve segmentation performance.

**Table 7:** Segmentation results of different methods on DRA, black bold represents the highest value.

Dataset	Search rules			IoU(%)	Dice(%)	Recall(%)	Specificity (%)	Precision (%)
	Similarity measure		Search area					
	$d_f$	$d_s$	global					
GlaS	$\checkmark$	$\times$	$\checkmark$	81.89	89.90	89.93	89.63	90.25
	$\checkmark$	$\checkmark$	$\times$	82.45	90.23	90.69	89.42	90.11
CoCaHis	$\checkmark$	$\times$	$\checkmark$	68.81	81.37	89.01	86.38	75.27
	$\checkmark$	$\checkmark$	$\times$	70.24	82.45	86.95	88.90	78.60

#### Results of different parameters on DRA

**Figure 12:**
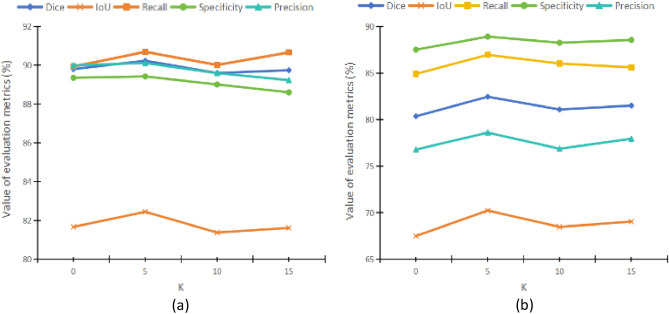
When using DRA, the impact of different k values (**a**) on the GlaS dataset and (**b**) on the CoCaHis dataset.

We conduct multiple groups of experiments on the parameters used in the training process to ensure that the selected parameters can make the model achieve the best effect. The main parameter in the module is the number of region partitions k. K represents the number of partitions, which controls the size of the module’s perception area range. In the experiment, we will choose different values of k for the experiment, and each change in k will increase by 5 to find the optimal value of k. The specific experimental results are shown in Fig. 12.

From Fig. 12, it can be seen that the model exhibits the best segmentation performance when using the DRA module at k = 5. From the results of the GlaS dataset, we can see that the smaller the number of region partitions, the better the effect. This may be due to the fact that the smaller the number of region divisions in the feature map, the more feature information is present in the divided regions, and the more feature data is used to represent each region. Therefore, each pixel in the region can interact with more similar pixels around it, reducing the possibility of information loss. In the results of the CoCaHis dataset, we can see that selecting the appropriate number of region partitions k has a significant impact on the improvement of the module. Due to the complexity of the foreground information in this dataset, the value of the number of regions divided, k, is crucial. But compared to not adding the DRA module, the DRA module has an improvement effect on the segmentation accuracy of the model in any k value.

## Conclusions

In this paper, an adaptive feature extraction and region-level information fusion medical image segmentation network is proposed. It uses CNN as the backbone for semantic feature extraction. Noticed that the targets of medical images usually exist in regions, we designs a dynamic regional attention module, which uses a similarity measure to extract features. Different from local fixed or global forms, this method can adaptively select a suitable modeling range based on features. At the same time, we also use the ordered shift MLP module to enhance the feature interaction within different regional blocks, so that the network can not only focus on the fixed size of the region, but also focus on the feature information of the more distant or surrounding areas. In this way, we can enhance the network’s ability to extract local detail information and reduce information loss, thereby more accurately and reliably segmenting the target area in medical images. After in-depth analysis, we have found reasonable parameters to achieve better experimental results. The experimental results show that the proposed method performs better on the dataset than other methods. It not only breaks the limitation of using local fixation to extract information, but also brings a new solution for dealing with data with scattered foreground information. Compared with other methods, we have better results in model parameters and computational complexity, but our inference speed is slightly slower than some methods. So, our future work will not only focus on extending this multi angle feature extraction method to other medical images, but also improve the running rules of the algorithm to achieve higher efficiency.

## Data Availability

Two public datasets, GlaS and CoCaHis were used to support this study and are available at https://doi.org/10.1016/j.media.2016. 08.008 and https://doi.org/10.1016/j.bspc.2020.102402. These prior datasets are cited at relevant places within the text as Refs.^48,49^.
